# Integration of GIS, Electromagnetic and Electrical Methods in the Delimitation of Groundwater Polluted by Effluent Discharge (Salamanca, Spain): A Case Study

**DOI:** 10.3390/ijerph14111369

**Published:** 2017-11-10

**Authors:** Rubén Vidal Montes, Antonio Miguel Martínez-Graña, José Ramón Martínez Catalán, Puy Ayarza Arribas, Francisco Javier Sánchez San Román, Caridad Zazo

**Affiliations:** 1 Department of Geology, Faculty of Sciences, University of Salamanca, 37008 Salamanca, Spain; r11vidal@usal.es (R.V.M.); jrmc@usal.es (J.R.M.C.); puy@usal.es (P.A.A.); javisan@usal.es (F.J.S.S.R.); 2 CSIC-National Museum of Natural Sciences, Geology Section, 28006 Madrid, Spain; mczc65@mncn.csic.es

**Keywords:** pollution, groundwater, GIS, geophysics, effluent discharge

## Abstract

The present work envisages the possible geometry of a contaminated plume of groundwater near hospital facilities by combining GIS (Geographic Information System) and geophysical methods. The rock underlying the soil and thin sedimentary cover of the study area is moderately fractured quartzite, which makes aquifers vulnerable to pollution. The GIS methodology is used to calculate the area that would be affected by the effluent source of residual water, based on algorithms that consider ground surface mapping (slopes, orientations, accumulated costs and cost per distance). Geophysical methods (electromagnetic induction and electric resistivity tomography) use changes in the electrical conductivity or resistivity of the subsurface to determine the geometry of the discharge and the degree of contamination. The model presented would allow a preliminary investigation regarding potential corrective measures.

## 1. Introduction

Water is a vital resource for human life, and therefore its management has to be rational and sustainable. Currently, in developed countries, surface and underground water resources are the focus of studies that analyses its potential for contamination. The problem in these cases is not related to finding water resources, but to find good quality resources. Water quality in a region depends on natural processes that generate, transport, and store it (e.g., precipitation rate, weathering processes, geochemistry of rock store aquifers). Accordingly, human activities, which are likely to contaminate this resource (e.g., urban industrial activities, agricultural activities, exploitation of water resources), should be controlled.

The discharge of sewage into land is one of the main causes of water pollution. To avoid this contamination, anthropogenic activities and their effluents should be located in areas resilient to water pollution (impermeable rocks, clay-like geological materials, etc.). In order to know the potential danger of contamination, vulnerability maps help to plan different land uses and to find the best location for human activities [1,2,3]. Although European environmental legislation requires the analysis of such risks (environmental assessment procedures: strategic and impact) this only applies to start-ups. In addition, many activities were developed prior to these standards and are still active. It is therefore necessary to apply extant or new methodologies to establish the possible contamination of water by different activities or pollution sources [4,5,6,7,8,9,10,11,12,13]. 

The aim of this work is to model the leachate generated by human activities (wastewater) from the Los Montalvos hospital, a facility close to Salamanca, and not connected to any urban sewer system. GIS methods were used to estimate the spatial extent of the contaminated area. GIS provides geospatial analysis tools, which integrate thematic maps (slopes, flow accumulations, orientation) in the correct determination of the slope and effluent discharge of wastewater.

For the detection of the extent of water pollution by effluent discharge, we used electromagnetic and electrical methods [12,13,14,15,16,17,18,19,20,21,22,23,24,25,26]. This case study shows that potentially polluting leachates from human activities can be investigated by easy to implement, low-cost techniques [27,28,29].

### Study Area and Geological Context

**Figure 1:**
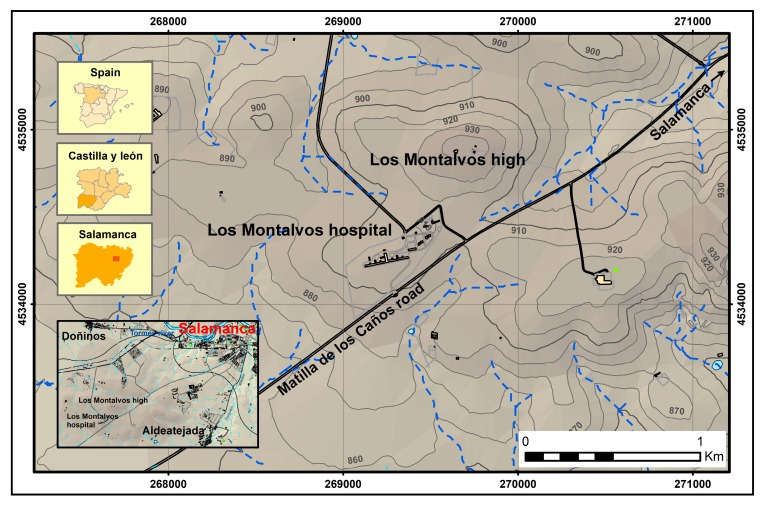
Study area and topographic map (dashed lines: drainage network).

The study area is located to the SW of Salamanca (Figure 1). From the geological point of view, the substrate is composed of Cambrian and Ordovician met sediments (shales and siltstones of the Aldeatejada Formation, the Armorican Quartzite and Middle Ordovician slates), partially covered by Neogene sediments and red detrital Quaternary surface formations (alluvial fans, glacis, colluvial and alluvial valley floor). The hospital under study is located on these low permeability lithologies, which present significant fracturing, originating aquifers with hydraulic conductivities ranging between 10^−5^ and 10^−2^ m·s^−1^ [2]. In the area covered by the geophysical survey, moderately fractured quartzite underlies a thin colluvial deposit made of clay and quartzite pebbles. The quartzite layers are folded in a wide E-W trending syncline with second order folds whose limbs dip moderately to strongly to the N or NE and S or SW.

**Figure 2:**
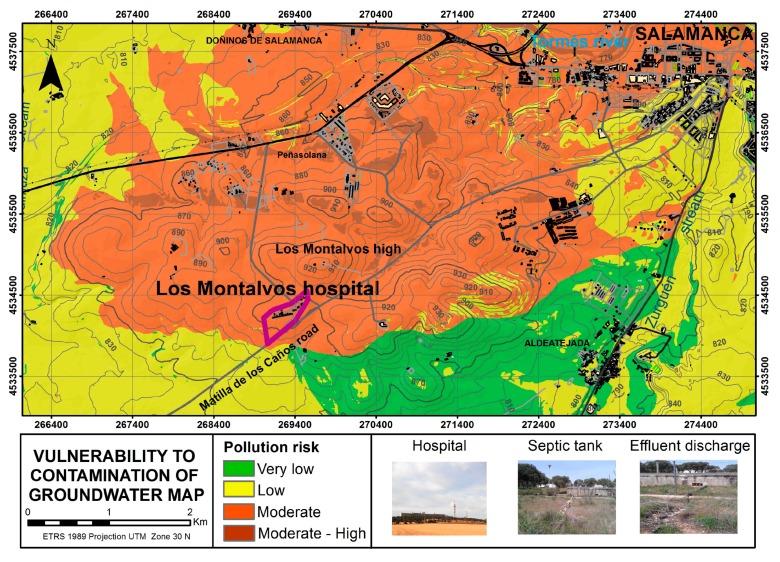
Vulnerability map [3].

The geometry of the aquifers is complex and difficult to detect, since it depends on inter-connecting fissures and/or cracks [3]. The hospital septic tank is made of concrete. Waste water from the hospital is treated inside this tank. The less polluted treated water is spilt onto the ground, where it is cleaned through filtration. The residual water is poured onto the ground outside the wire fence enclosure. The study area has a moderate degree of vulnerability to the contamination of groundwater, as concluded after applying the DRASTIC method (Figure 2), so that discharges can adversely affect the water quality and contaminate aquifers [14,15,16,17,18,19,20,21].

## 2. Methodology

In this study, we used a two-step approach: a first phase of spatial modelling techniques to delineate the area affected by pollution was followed by a second phase of conductivity/resistivity data acquisition and modelling designed to show the distribution and extent of contamination in the study area.

### 2.1. Application of GIS Techniques

**Figure 3:**
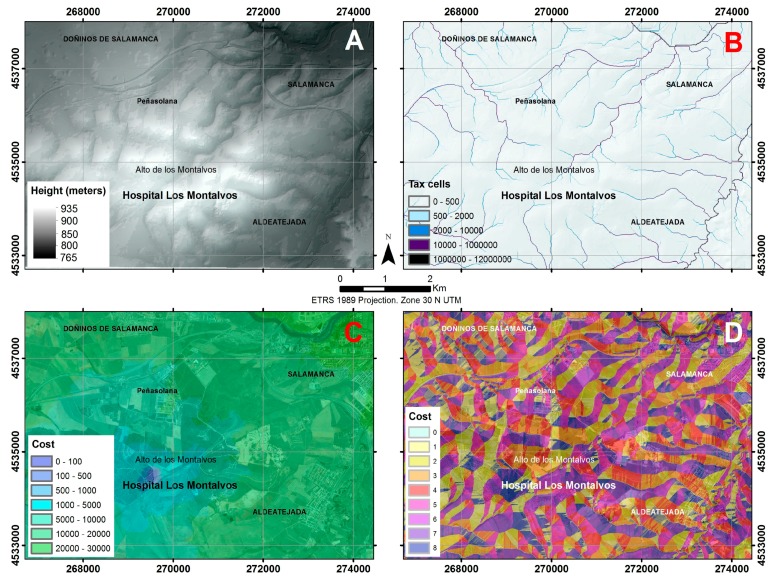
Methodological sequence with GIS (Geographic Information System) technique: digital elevation model (**A**), drainage network (**B**), accumulated costs (**C**) and possible routes map (**D**).

GIS methods (Figure 3) aim to predict the behavior of the emission of an effluent discharge from the septic tank to the ground, using scripts integrated in ArcGIS v.10.3 (ESRI Inc., Redlands, CA, USA). The results of this prediction will serve as the basis for the design of a geophysical prospecting strategy.

To determine the pollutant route, a spatial analysis was conducted using the cost distance algorithm in ArcGIS. The route of least cost was calculated using pixel values derived from the slopes in the digital elevation model. The cost distance algorithm is similar to the Euclidean function, but instead of calculating the actual distance from one point to another, it calculates the weighted shortest distance (i.e., the accumulated travel cost). The route chosen as the case study is newly designed. The units used are not geographical units but cost units, in this example, units of significant pollutant features. Through the process of calculating the cumulative travel cost, a route map was generated (Figure 3). During the map generation process, each grid cell was selected in relation to the nearest source pixel. The travel cost value was obtained by multiplying the size by the value of each pixel, which indicates the environmental cost of that pixel calculating the cumulative costs for travel. The potential cumulative costs of a route are calculated by totalling the distance, assuming that the centre of each pixel is a point that can be connected to the surrounding pixels. Distance can occur either between adjacent pixels, for example between pixel one (cost1) to a pixel two (cost2), determining the total cost of moving (D1) empirically (Equation (1)), or between pixels whose displacement is diagonal (Equation (2)).

D1 = (cost1 + cost2)/2(1)

D1 = 1.414214 (cost1 + cost2)/2(2)

The cumulative cost calculation for the movement between pixels (Equation (3)) is performed such that the cost of travelling between three pixels (i.e., cost1, cost2 and cost3) is the sum of “D1” (Equation (1)) and “D2” (Equation (4)) and so on until “Dn”.

AC = D1 + D2 +…. + Dn,(3)

D2 = cost2 + cost3/2(4)

To predict the route and the direction depends on the spatial orientation of travel between the origin point and the end point. The previously-described calculation of accumulated costs for each pixel provides the cumulative costs for the route. The calculation of route direction is determined for each pixel as the direction the route must travel (i.e., the next cell that they must pass through) to reach the point of arrival at the lowest possible cumulative cost. This calculation is achieved by assigning to points of departure and arrival a value of zero and values to the adjacent pixels of one to eight. The adjacent pixels are assigned their respective values by proceeding in a clockwise manner from pixel zero as follows: right (1-yellow), lower-right (2-green), down (3-pale blue), lower-left (4-dark blue), left (5-very dark blue), upper-left (6-pink), up (7-red) and upper-right (8-orange).

Using the values of accumulated cost and route direction, the lowest cost path from a given departure point (hospital) to a given arrival point (nearby streams) can be determined. This pollutant route is determined by using the “shortest path” (“least cost”) command in ArcGIS, which automatically links the lowest pixel values with respect to adjacent pixels.

**Figure 4:**
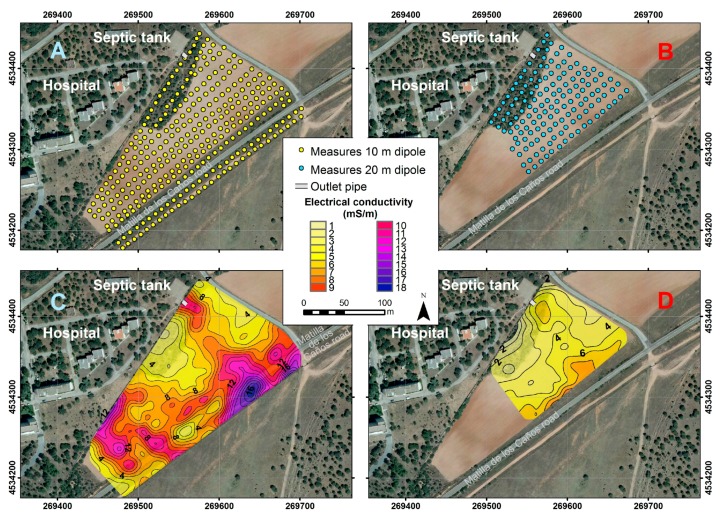
Meshing of sampling points for the separation between the coils of 10 m (**A**) and 20 m (**B**). Electrical conductivity maps obtained with a horizontal dipole (vertical coils) with a spacing of 10 m (**C**) and 20 m (**D**).

First, all seasonal and permanent streams in the area were identified, since they will be the destination of any spillage travelling on the earth’s surface [3]. To do this, the digital elevation model (DEM) of the study area, with a spatial resolution of five meters (Figure 3A), was modified by filling all possible sinks to get depression areas at the same height as the surrounding terrain so the fluids can reach topographically lower areas. Then, with the corrected DEM, a flow direction mapping was created from the slope mapping, allowing us to find the direction that the effluent has in each pixel (since the flow direction is conditioned by the slope at each point). Following this, the mapping of the drainage network was carried out with the raster calculator by imposing a need of at least 500 tributary upstream cells (among the ones previously calculated) in each pixel (Figure 3B). Once the drainage network was known, a map of accumulated costs was performed, to define the streams that are most likely to be affected by effluent discharge coming out of the septic tank. This tool (Figure 3C), analyses the degree of difficulty for the fluid to move through the ground downslope. It uses the relative distances between the origin determined by the point of effluent discharge and the end point, which are nearby streams. Accordingly, a new map was generated which establishes links of shortest possible routes (Figure 3D), reflecting the difficulty for water to move from the source to any channel. The higher the value of the cost, the greater the difficulty to get the spill in that cell. Thus, the cost is zero in each of the cells that define the streams. Finally, with GIS techniques, we determined the possible contamination zone generated by the effluent discharge of the studied human activity (Figure 4) [3]. 

### 2.2. Application of Electromagnetic and Electric Methods

The geophysical techniques used for this work are electromagnetic induction and electric tomography. Both contribute to delimiting the geometry of the contaminated area, associating the effluent influence with locations with higher electrical conductivity (lower resistivity). Electromagnetic induction has the advantage of providing conductivity maps at variables (always shallow) in a continuous area over a wider space than ERT (Electrical Resistivity Tomography). The system used in this study was EM34-3 of Geonics Limited (Geonics Limited, Mississauga, ON, Canada). It is composed of two coils (transmitter and receiver) with their respective consoles; two wires for connection between the rings and its box; and a link wire between boxes of different lengths (10 and 20 m).

The electromagnetic induction (EM) technique is based on a sinusoidal alternating current of frequency f, provided by the transmitter, which causes a primary electromagnetic field Hp that penetrates the subsurface and the surface at a depth that depends of the distance between the coils. If a conductive body exists, the magnetic component induces electric currents in that body, which in turn generates a secondary magnetic field Hs that is recorded by the receiver. The ratio between the primary and secondary fields provides an estimation of the conductivity of the subsurface at the midpoint between the coils and at a depth that depends on the distance between them and their orientation (vertical or horizontal). The calculated conductivities are apparent, since they represent average values for the whole area. To obtain electrical conductivity maps, a grid pattern must be sampled. The values forming the conductivity map are the mid-points between the coils at each position. 

The acquisition was carried out along two grids of 10 m × 10 m, one with a coil distance of 10 m and the other of 20 m. With both grids, two datasets were acquired: one with horizontal coils or loops (HLEM), which create a vertical magnetic field, and the other with the coils in a vertical position (VLEM), which implies a horizontal magnetic field.

There is a concrete and metal fence limiting the hospital area, adjacent to the westernmost NE-SW line of measurements of the survey area (Figure 4A). Also, a barbed-wire fence exists to the SE, adjacent to the southernmost line of measures, and a buried gas conduit possibly exist parallel to this second fence, between it and the ENE-WSW road.

The use of vertical coils provided better results, which are shown in Figure 4. The maps acquired with horizontal coils have a great influence on the conducting objects (fences and possible gas conduit), and are not shown. The 10 m grid (Figure 4A) covers an area larger than the 20 m area (Figure 4B). Measurements were made in the dry season, allowing at least five days since last rainfall and the day of data collection, to avoid influencing the results.

EM contour maps were created using a spline interpolation of the data measured in the field. Interpolation estimates the values of unknown points using mathematical functions that fit and pass through the nearest input points, presenting minimum curvatures, taking into account that the sum of the squares of the terms of the second derivative taken on each point on the surface should be minimal. This method is suitable in cases where the amount of data used to interpolate is high, resulting in surfaces which vary very slightly. ArcGIS allows the use of two types of splines: regularized and tension. In the regularized spline, the created surfaces are smoothed and gradually change the values that can be found outside the range of sample data. Contrarily, the tension spline type can control the rigidity of the obtained surfaces according to the modelling phenomenon, generally obtaining less smooth surfaces, with values more limited by the range of sample data. In our study, we used regularized spline, since no major changes in the various weather parameters, or electrical conductivity maps were expected.

The ERT method provides electrical resistivity cross-sections along user-designed profiles. The resulting models can be integrated with electromagnetic induction results providing detailed information of highly resistive areas where secondary electromagnetic fields are not significant. In this case we used the PASI 16SG24-N system (PASI srl, Torino, Italy). It consists of a central station (resistivimeter) powered by a 12 V battery, an energizer (control-system-induced electrical signal), and a link box for each of two cables and connections for sixteen electrodes each. The latter are made of stainless steel which, together with multiple measurements reversing direct current polarity, minimizes electrode polarization.

Electrical tomography is based on acquiring values of the apparent resistivity along one or more profiles in a redundant way, i.e., using all the possible combinations between equally-spaced electrodes. To measure the apparent resistivity at each point, the resistivimeter uses four electrodes at a time: two current electrodes, which introduce electric power in the ground, and two detection or potential electrodes, from which the potential difference is measured. We used a Wenner configuration, since it provides a better vertical resolution together with a reasonably good horizontal resolution. This configuration is centered and characterized by a spacing between the current electrodes three times greater than that of potential electrodes. Among all possible configurations, the Wenner provides the better resolution for horizontal structures and flat bodies.

The output of the resistivimeter is a pseudosection of apparent resistivity that requires an inversion procedure to calculate a realistic model of the subsurface electric properties in the chosen vertical section [30,31,32]. For inversion, we have used the finite-element-based RES2INV software (Geomatrix Earth Science Ltd., Leighton Buzzard, UK). The resulting model reaches a depth that depends on the length of the profile; in our case 124 m, which with the Wenner configuration reaches 25 m deep in the central part. 

**Figure 5:**
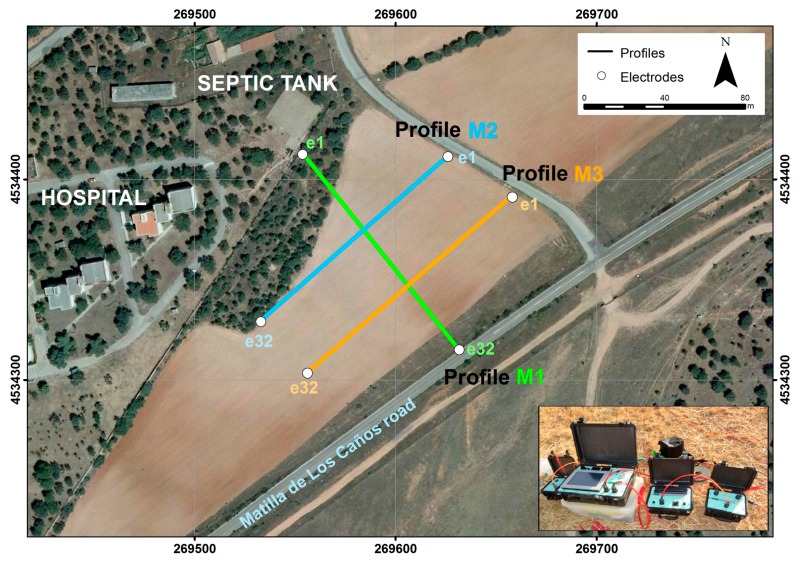
A. Location of acquisition of three profiles M1, M2 and M3. The inset shows the PASI 16SG24-N resistivimeter, the energizer and the two link boxes.

Three profiles—M1, M2 and M3—were acquired and inverted (Figure 5). M1 starts at the outlet pipe of the hospital septic tank (e1), and runs down slope striking NW-SE, following the trend of maximum conductivities obtained with electromagnetic induction (Figure 4C,D). The other two profiles are perpendicular, and cross M1 at 1/3 (M2) and 2/3 (M3) of its length approximately.

## 3. Results and Discussion

**Figure 6:**
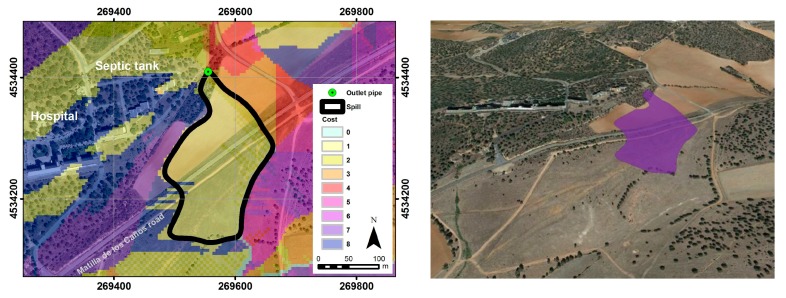
Delimitation of effluent discharge using GIS techniques based on a cost–distance algorithm (**left**) and 3D (three dimensional) modeling with an ArcScene script (**right**).

Using the “shortest route distance map” utility and taking into account the calculated flow directions from the slopes of DEM, the shape of the discharge area was modelled, obtaining a surface condition of 2.9 ha (280 m long and ~190 m wide). The area in 3D (three dimensional) shows the affected farmland located to the south of the hospital, in front of the septic tank area (Figure 6).

The results of the VLEM method (Figure 4C), represent values of conductivity with a very strong influence of the bodies located at the surface. For 10 m of coil separation, the maximum contribution is from the surface, the maximum research depth is 7.5 m, and the depth of the optimum contribution is 3 m. Figure 4 shows that from the outlet of the septic tank, there is a region with high conductivity values up to σ = 10 mS·m^−1^, which is elongated in the slope direction (NW-SE), with a trend similar to that shown by GIS techniques. This area increased its maximum to the south, where its trend changes to NNE-SSW, as in the GIS map, reaching values of σ =18 mS·m^−1^. However, the latter feature may be partially enhanced by the gas pipeline whose exact position is unknown, but whose output is at the northern edge of the ENE-WSW road. Other possible contributors may be the reinforced concrete poles of the fence, and perhaps buried metal ducts linking both sides of the road to allow surface water runoff. In the southwestern section of the map (Figure 4C) there is also an area with high values of conductivity that is partly influenced by the fence of the hospital, which is located immediately to the north. The remaining areas, with conductivities lesser than σ = 5 mS·m^−1^ reflect the presence of quartzite with less water or, more probably, less conductive contaminant content.

The VLEM analysis with 20 m separation between coils (Figure 4D) reflects conductivity values until a maximum depth of 15 m, with a strong maximum contribution of the surface materials, and an optimum depth of investigation of 6 m. In this case, the anomaly shows similar behavior to that described above, but with a clear decrease in conductivity values, implying that the spill imaged by the EM method is very superficial. This would not exclude another part of the spill occurring at more than a few meters deep.

**Figure 7:**
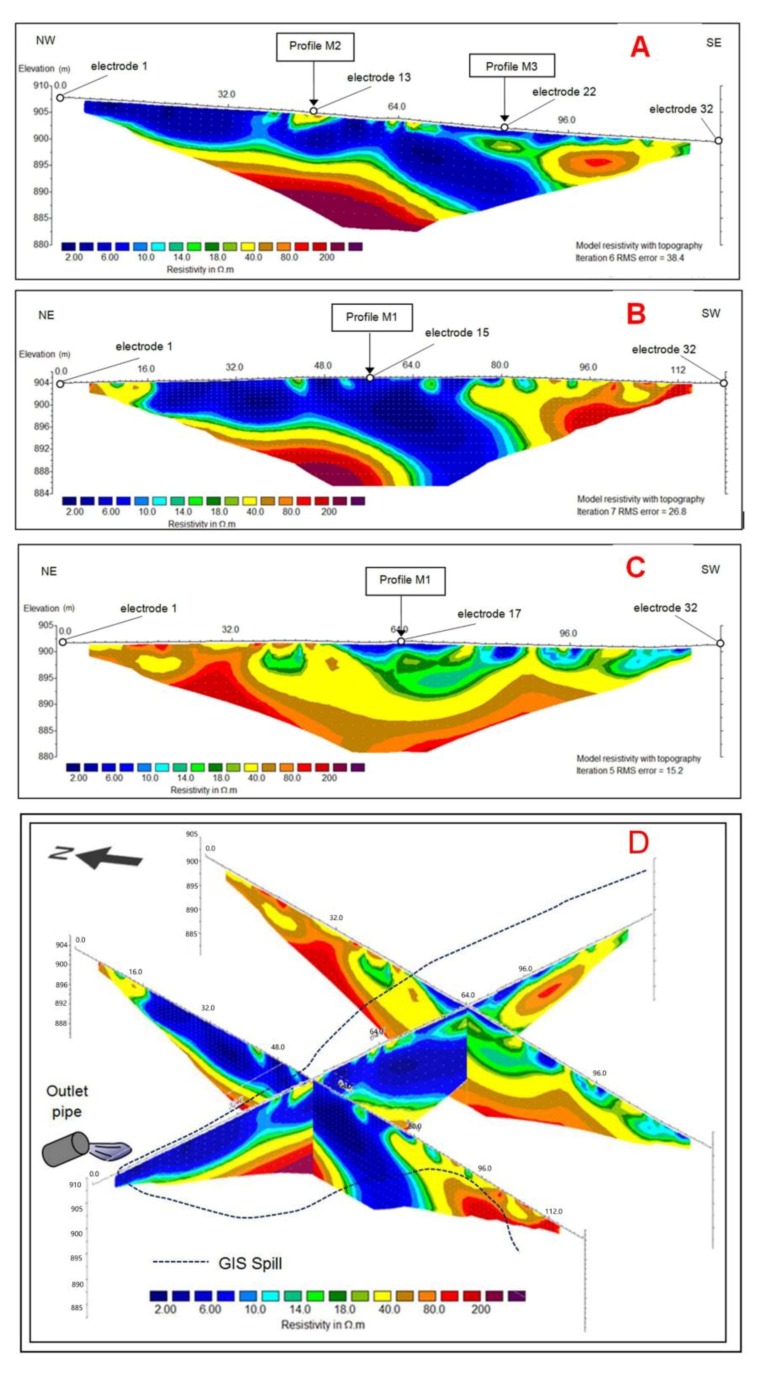
ERT (Electrical Resistivity Tomography) profiles M1 (**A**), M2 (**B**) and M3 (**C**), and 3D layout (**D**). The surface limit of the spill as deduced from GIS methodology is included (dashed line).

Fortunately, the ERT profiles show higher resolution results of the geometry of the spill. The profiles acquired (Figure 5) are shown in Figure 7: Profile M1 strikes NW-SE and goes from the septic tank to the road (Figure 7A), and the other two are transverse profiles M2 (Figure 7B) and M3 (Figure 7C). A 3D visualization of the three profiles (Figure 7D) allowed the correlation of areas of high conductivity, with resistivity values lower than 12 Ω·m (blue colors), from others with higher resistivity (warmer colors), probably regions where the accumulation of effluent is limited or absent.

**Figure 8:**
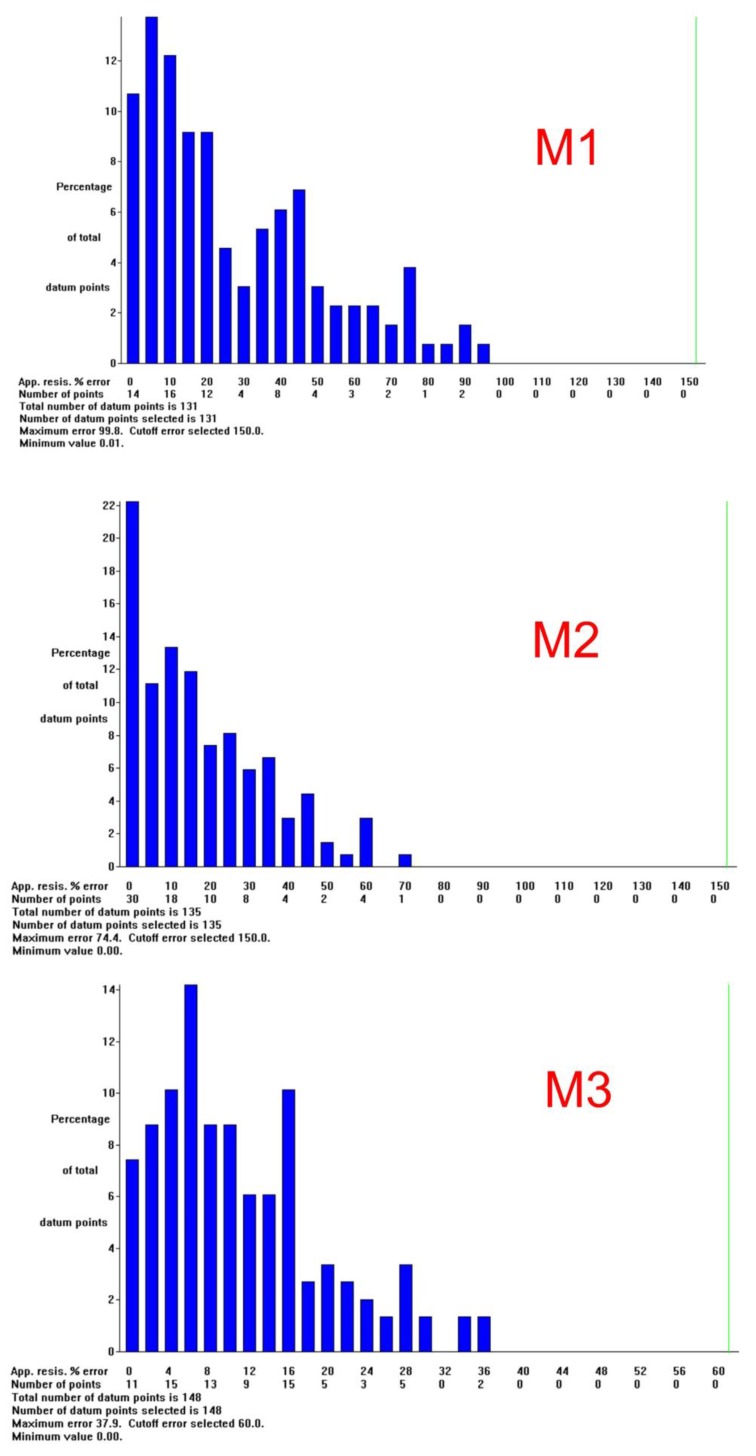
Bar chart of RMS (Root Mean Square) error statistics for the three profiles.

The whole RMS (Root Mean Square) error (in %) is shown at the lower right corner of each profile. Figure 8 depicts a bar chart of RMS error statistics for the three profiles. It shows the percentage of difference between the measured and calculated apparent resistivity for the corresponding iteration. Each bar indicates the number of points for which the error is between a given interval, with these divided into steps of 5%.

In profile M1 (Figure 7A), the surface area affected by the spill covers a length of about 112 m from its source, but at 72 m, the low resistivity anomaly splits in two, a superficial strip 1–3 m thick and a broad band, 10–12 m thick, that dips to the SE and reaches a depth of more than 15 m. The initial meters of the section show that the penetration of the injected fluid from the outlet pipe is low, around 3 m, and as it moves in favour of the slope, it gains penetration, featuring a thickness of up to 7 m below the surface. In the SE part of the section (beyond 72 m), the spill appears to follow a steeper path in the direction of the descending slope, probably reflecting a layer of relatively porous or heavily-fractured quartzite. The upper part of the low resistivity anomaly images shows the water discharged from the hospital complex and soaking the soil and thin sedimentary cover of the farmland, and fits the discharge as deduced from GIS and EM methods. The lower, thicker part reflects infiltration into the basement, and would have remained undetected without the ERT survey. Profile M2 is similar to M1 except for the continuation of the superficial thin band of low resistivity beyond 80 m, although low resistivity spots occur until meter 98 (Figure 7B). Intersecting the M1 profile at 48 m from the spill source in the NW-SE direction, the region under the influence of the spill has a width of 65 m and vertically affects a minimum thickness of 7 m to the NE, and up to 17 m in the area to the right of middle, where the spill seems to enter into the basement with a dip of 30–40°. Again, this part of the potentially contaminating plume would have remained unknown with only the GIS and M surveys.

Two aspects deserve to be highlighted from the comparison between profiles M1 and M2. The first is that the superficial part of the discharge, as deduced by the GIS method, is reasonably well-matched by the ERT profiles, as the anomaly is rather continuous in M1 and discontinuous in M2. The latter is what could be expected at the boundary of the modelled effluent discharge, whose NW limit is close to the second profile (Figure 6 and Figure 7D). The EM maps also fit the resistivity distribution of the ERT profiles reasonably well, except at the far end of M1, where the low resistivity measured by the VLEM method does not appear. This is a strong argument in favour of the existence of buried metallic objects in the SE limit of the survey area, close and parallel to the road, and out of the reach of profile M1.

The second aspect is that from the two profiles, it can be deduced that the highly conductive band where the spill seems to infiltrate deeper into the basement is roughly E-W, coinciding with the attitude of the quartzite layers.

Profiles M1 and M2 match within the uncertainty calculated by the RMS error at the intersection. Only the upper 2 m of relatively high resistivity in M1 do not appear at the crossing point in M2. This is not surprising, because the vertical line under the intersection reflects measurements made with electrodes placed at some distance from that point, but aligned in the NW-SE and NE-SW directions, respectively. Another cause of mismatch is inversion. Each profile is inverted independently, and tries to fit its apparent resistivity measured in a particular direction in a model of supposedly real values without much control of actual resistivity at both sides of the profile. Furthermore, high contrasts and complex distribution of the measured apparent resistivity were found by the inversion procedure, and are reflected in the high values of the RMS error, which has a value of 38.4 in M1 and 26.8 in M2 (Figure 7A,B). This means that the inverted distribution should not be taken as the real one, but as an approximation, and comparing two approximations should not provide an exact fit.

The lower limit of the low resistivity region in M1 and M2 matches, if not perfectly, surprisingly well, given the fact that for the deep part of the profiles, the electrodes involved in the acquisition are far from the intersection, and it can be expected that the rocks encountered by the flow lines of the induced electric field could differ in electrical properties.

Profile M3 intersects M1 at 84 m from the spill source in the NW-SE direction (Figure 7C). It shows that the spread of pollution at the surface continues to the south, as it is similar to that of the southern part of M1, and in agreement with the GIS and VLEM data (Figure 4C,D and Figure 7D). The RMS error is 15.2, the lesser of the three profiles, and down to 5 m deep, it fits M1 at the intersection. Deeper, however, the agreement is poor, although a band with relatively low resistivity values (12–18 Ω·m) reaches a height of 900 m above sea level, which loosely compares with the 885 m or less height of the wide and inclined band in M1, which is characterized by a lower resistivity (less than 10 Ω·m; Figure 7A). The band in M3 might show the same layer folded in an open syncline, whose south-dipping northern limb is clearly shown in M1 and M2.

## 4. Conclusions

The multidisciplinary approach used in this work, combining GIS, EM and ERT, shows an effective, low-cost and non-invasive methodology that can be helpful in the estimation of contamination generated by human activities.

The presented GIS method provides a feasible estimation of the extent of groundwater contaminating plumes close to the surface, as proved in our case study of sewage discharge near a hospital facility. It provides useful information regarding where to carry out further studies applying geophysical methods. The geophysical techniques confirmed the conclusions reached with the GIS tools, and provide further insights into the geometry of the discharge from the surface at depths of a few to tens of meters. In particular, electric resistivity tomography (ERT) proved successful in following the infiltration into basement rocks. In our case, the geometry of the contaminated body seems related to the structure of the folded layers, although a 3D survey would be necessary to check this possibility.
